# Identification of Oral Microbiome Biomarkers Associated with Lung Cancer Diagnosis and Radiotherapy Response Prediction

**DOI:** 10.3390/pathogens14121294

**Published:** 2025-12-16

**Authors:** Xiaoqian Shi, Nan Bi, Wenyang Liu, Liying Ma, Mingyang Liu, Tongzhen Xu, Xingmei Shu, Linrui Gao, Ranjiaxi Wang, Yinan Chen, Li Li, Yu Zhu, Dan Li

**Affiliations:** 1 State Key Laboratory of Molecular Oncology, National Cancer Center/National Clinical Research Center for Cancer/Cancer Hospital, Chinese Academy of Medical Sciences and Peking Union Medical College, Beijing 100021, China; shi18377936351@163.com (X.S.); mlyshiyi2009@sina.com (L.M.);; 2 Department of Radiation Oncology, National Cancer Center/National Clinical Research Center for Cancer/Cancer Hospital, Chinese Academy of Medical Sciences and Peking Union Medical College, Beijing 100021, China; binan_email@163.com (N.B.); liuwenyang@cicams.ac.cn (W.L.);; 3 Department of Clinical Laboratory, National Cancer Center/National Clinical Research Center for Cancer/Cancer Hospital, Chinese Academy of Medical Sciences and Peking Union Medical College, Beijing 100021, China; 4 Laboratory Animal Center, National Cancer Center/National Clinical Research Center for Cancer/Cancer Hospital, Chinese Academy of Medical Sciences and Peking Union Medical College, Beijing 100021, China

**Keywords:** oral microbiota, lung cancer, diagnostic model, treatment response prediction, radiotherapy

## Abstract

The oral cavity acts as the anatomical gateway to the respiratory tract, sharing both microbiological and pathophysiological links with the lower airways. Although radiotherapy is a cornerstone treatment for lung cancer, reliable oral microbiome biomarkers for predicting patient outcomes remain lacking. We analyzed the oral microbiome of 136 lung cancer patients and 199 healthy controls across discovery and two validation cohorts via 16S rRNA sequencing. Healthy controls exhibited a significantly higher abundance of *Streptococcus* compared to patients (*p* = 0.049, *p* < 0.001, *p* < 0.001, respectively). The structure of the microbial community exhibited substantial dynamic changes during treatment. Responders showed enrichment of *Rothia aeria* (*p* = 0.027) and *Prevotella salivae* (*p* = 0.043), associated with prolonged overall survival (OS) and progression-free survival (PFS), whereas non-responders exhibited elevated *Porphyromonas endodontalis* (*p* = 0.037) correlating with shorter OS and PFS. According to Analysis of Compositions of Microbiomes with Bias Correction 2 (ANCOM-BC2) analysis, *Akkermansia* and *Alistipes* were nearly absent in non-responders, while *Desulfovibrio* and *Moraxella* were virtually absent in responders. A diagnostic model based on *Streptococcus* achieved area under the curve (AUC) values of 0.85 (95% CI: 0.78–0.91) and 0.99 (95% CI: 0.98–1) in the validation cohorts, and a response prediction model incorporating *Prevotella salivae* and *Neisseria oralis* yielded an AUC of 0.74 (95% CI: 0.58–0.90). Furthermore, in small cell lung cancer, microbiota richness and diversity were inversely correlated with Eastern Cooperative Oncology Group (ECOG) performance status (*p* = 0.008, *p <* 0.001, respectively) and pro-gastrin-releasing peptide (ProGRP) levels (*p* = 0.065, *p* = 0.084, respectively). These results demonstrate that lung cancer-associated oral microbiota signatures dynamically reflect therapeutic response and survival outcomes, supporting their potential role as non-invasive biomarkers for diagnosis and prognosis.

## 1. Introduction

Lung cancer is the leading cause of global cancer incidence and mortality according to Global Cancer Statistics (GLOBOCAN 2022). An estimated 1.8 million deaths were attributed to lung cancer in 2022, accounting for 18.7% of global cancer mortality [1]. Lung cancer is primarily categorized into non-small cell lung cancer (NSCLC) and small cell lung cancer (SCLC), with NSCLC constituting over 85% of all cases. Distant metastasis is present in 48% of patients at diagnosis, associated with a 5-year relative survival rate of only 8%. In contrast, patients with localized disease exhibit a significantly higher 5-year survival rate exceeding 60% [2]. Consequently, early diagnosis is critical for improving lung cancer prognosis.

The oral microbiota, the second largest microbial community in the human body, comprises over 770 bacterial species [3]. Given the anatomical and physiological continuity between the oral cavity, upper respiratory tract, and the lungs, the potential influence of the oral microbiome on lung cancer pathogenesis is increasingly recognized. Recent investigations reveal a pronounced enrichment of specific *Neisseria* and *Actinomyces* species in the saliva of responders with advanced NSCLC, suggesting their utility as potential predictive biomarkers for immune checkpoint inhibitors efficacy [4]. While current lung cancer management integrates multidisciplinary team (MDT) consensus and personalized treatment strategies, incorporating surgical resection, radiotherapy, systemic chemotherapy, targeted therapy, and immunotherapy. It is noteworthy that radiotherapy remains a primary treatment for locally advanced lung cancer; however, predictive oral microbial biomarkers for radiotherapy response are currently lacking.

This study investigates the composition and temporal dynamics of the oral microbiome in lung cancer patients compared with healthy controls through 16S rRNA sequencing and subsequently validates the key findings against independent validation cohorts from public databases. It aims to identify microbial signatures associated with lung cancer diagnosis and therapeutic outcomes, elucidate their potential functional roles, and thus provide novel biomarkers for diagnosis and treatment response prediction.

## 2. Materials and Methods

### 2.1. Study Participants

From April 2021 to March 2022, we conducted a real-world study on comprehensive treatment of lung cancer based on precision radiotherapy at the Cancer Hospital, Chinese Academy of Medical Sciences (CAMS). During this study, twenty-four patients with histologically confirmed lung cancer were enrolled. Thirty-eight eligible healthy controls were individuals with normal results on annual health examinations conducted within the study recruitment period, including chest X-ray, abdominal ultrasound, and blood tests. Collectively, the discovery cohort encompassed 24 lung cancer patients and 38 healthy controls. The study protocol was approved by the Ethics Review Committee of the Cancer Hospital, CAMS (No. 20/453-2649) on 30 December 2020.

All participants in this study (both patients and healthy controls) met the following criteria: (1) Absence of severe oral diseases or systemic conditions (e.g., acute oral ulcers, oral tumors). (2) No recent oral treatments (e.g., dental scaling, periodontal surgery) within 1–2 weeks prior to sample collection. (3) No intake of oral antibiotics or hormones within 3 months prior to sample collection. Patients enrolled in this study met the following criteria: (1) Histologically confirmed diagnosis of lung cancer without other concurrent malignancies. (2) All patients adhered to the planned complete volumetric modulated arc therapy regimen. All participants who agreed to be sample donors provided written informed consent.

In addition, we utilized publicly available 16S rRNA data of oral microbiota for external validation by incorporating datasets generated using the same sequencing platform and primers into a unified cohort. Validation cohort 1 comprised PRJEB44168 (91 lung cancer patients) [5] and PRJEB48982 (81 healthy controls) [6], while validation cohort 2 included PRJNA904049 (21 lung cancer patients) [7] and PRJNA822496 (80 healthy controls) [8]. In total, the validation cohorts encompassed 112 lung cancer patients and 161 healthy controls. Detailed information on validation cohorts is presented in Table S1.

### 2.2. Sample Collection and Storage

Mouthwash samples were collected from the 24 lung cancer patients at two time points: before radiotherapy and after radiotherapy, yielding a total of 48 samples. Additionally, 38 mouthwash samples were collected from the 38 healthy control subjects.

Prior to sample collection, all participants were required to abstain from smoking, alcohol consumption, food intake, and use of oral hygiene products for at least 2 h. During collection, participants rinsed their mouths with approximately 30–50 mL of sterile physiological saline for 15–30 s. The collected mouthwash samples were transported to the laboratory within 1 h and stored at −80 °C for subsequent analysis.

### 2.3. Clinical Data Collection

We collected the following clinical data from patients: (1) basic information: age, height, weight, body mass index (BMI), Eastern Cooperative Oncology Group (ECOG) performance status score, Karnofsky Performance Status (KPS) score. (2) complete blood count: red blood cell (RBC), hemoglobin (Hb), white blood cell (WBC), neutrophil (Neut), lymphocyte (Lymph), monocyte (Mono), eosinophil (Eos), basophil (Baso), platelet (PLT). (3) biochemical indicators: alanine aminotransferase (ALT), aspartate aminotransferase (AST), lactate dehydrogenase (LDH), gamma-glutamyl transferase (GGT), adenosine deaminase (ADA), total bile acid (TBA), total bilirubin (TBIL), albumin (ALB), urea, creatinine (CRE), uric acid (URIC), glucose (GLU), total cholesterol (CHOL), C-reactive protein (CRP). (4) tumor markers: carbohydrate antigen 125 (CA125), cytokeratin 19 fragment (Cyfra21-1), neuron-specific enolase (NSE), squamous cell carcinoma antigen (SCC), carcinoembryonic antigen (CEA), pro-gastrin-releasing peptide (ProGRP).

### 2.4. 16S rRNA Gene Sequencing

Microbial genomic DNA was isolated from mouthwash samples using the FastPure Soil DNA Isolation Kit (MJYH, Shanghai, China), adhering strictly to the manufacturer’s protocol. DNA purity and concentration were assessed through electrophoretic analysis on 1.0% agarose gels and spectrophotometric quantification with a NanoDrop2000 system (Thermo Scientific, Waltham, MA, USA). Purified DNA was stored at −80 °C until downstream processing. The V3-V4 hypervariable regions of bacterial 16S rRNA genes were subsequently amplified via PCR on a T100 Thermal Cycler (BIO-RAD, Hercules, CA, USA), employing universal primers 338F (5′-ACTCCTACGGGAGGCAGCAG-3′) and 806R (5′-GGACTACHVGGGTWTCTAAT-3′) [9]. The V3-V4 region of the 16S rRNA gene was sequenced on the Illumina NextSeq2000 platform (Illumina, San Diego, CA, USA) using paired-end 2 × 300 bp sequencing by Majorbio Bio-Pharm Technology Co., Ltd. (Shanghai, China).

### 2.5. Bioinformatics Analysis

Raw FASTQ files underwent quality control processing via fastp (v0.19.6) [10], followed by paired-end sequence assembly using FLASH (v1.2.7) [11]. High-quality merged sequences were subsequently clustered into operational taxonomic units (OTUs) at 97% similarity threshold through UPARSE (v7.1) [12]. Taxonomic assignment of representative OTU sequences was performed against the SILVA v138 reference database with a minimum confidence cutoff of 0.7.

Alpha diversity metrics were computed from OTU tables utilizing Mothur v1.30.1 [13]. The α diversity indices included richness indices (Sobs and Ace) and a diversity index (Shannon). Beta diversity patterns were evaluated via partial least squares discriminant analysis (PLS-DA) to determine inter-group community dissimilarities.

Differential abundance analysis of bacterial taxa across experimental groups was performed using three complementary approaches. First, the Wilcoxon rank-sum test was applied to compare relative abundances of individual bacterial taxa between pairwise groups. Additionally, we employed the Linear Discriminant Analysis Effect Size (LEfSe) algorithm [14] (http://huttenhower.sph.harvard.edu/LEfSe, accessed on 19 May 2025) to identify taxa exhibiting significant abundance differences from phylum to species level, using thresholds of LDA score > 2 and *p* < 0.05 for biological effect size. Furthermore, Analysis of Compositions of Microbiomes with Bias Correction 2 (https://bioconductor.org/packages//release/bioc/vignettes/ANCOMBC/inst/doc/ANCOMBC2.html, accessed on 4 June 2025) (ANCOM-BC2) [15] was implemented to detect statistically significant taxa while accounting for structural zeros-defined as taxa completely absent in specific groups due to biological irrelevance rather than technical limitations.

To address potential confounding from clinical heterogeneity, we performed the Cox regression analysis in a two-step process. First, all clinical variables (e.g., histology, stage, treatment regimen) were screened for association with progression-free survival (PFS) using univariable models. Significant variables from this stage were then included as candidates in a final multivariable Cox proportional hazards model.

Variance inflation factors (VIF) were calculated for clinical parameters using the vif function in the car package (v3.1-3) (https://cran.r-project.org/web/packages/car/car.pdf, accessed on 15 July 2025). Variables with VIF values greater than 10 were excluded to reduce multicollinearity. Subsequently, associations between clinical indices and microbial community structure were evaluated using distance-based redundancy analysis (db-RDA) implemented in the vegan package (v2.5-3). Relationships between key clinical variables identified by db-RDA and alpha diversity indices were further quantified using regression analysis.

Random forest modeling and receiver operating characteristic (ROC) analysis were performed using the randomForest (v4.7-1.1) and plotROC (v2.3.0) packages in the R statistical software. This approach enabled efficient identification of the most significant microbial taxa for discriminating between sample groups, followed by construction of classification and prediction models. Model stability was evaluated via bootstrap resampling (20 iterations) with replacement, preserving the original sample size in each iteration. For every bootstrap sample, a random forest model consisting of 500 decision trees was constructed.

For the discriminatory features, survival analysis was conducted using the Kaplan–Meier method to estimate survival probabilities, with inter-group comparisons assessed via the log-rank test. Additionally, the surv_cutpoint function from the survminer package (v0.4.9) was employed to perform optimal cutoff point analysis based on overall survival (OS) and progression-free survival (PFS) data, determining the best threshold for stratifying prognostic outcomes. This framework was applied to identify microbial features associated with patient prognosis.

Functional metagenomic profiling was inferred through Phylogenetic investigation of communities by reconstruction of unobserved states 2 (PICRUSt2) (v2.2.0) [16] based on phylogenetically placed OTU representative sequences.

### 2.6. Follow up and Statistical Analysis

Long term follow-up was maintained for all patients. Patients demonstrating tumor progression or recurrence within the 24-month follow-up period were classified as non-responders (NR). Those without documented progression or recurrence events were designated as responders (R).

Statistical analyses were performed using SPSS 25.0 (IBM, Armonk, NY, USA), with a two-tailed *p* < 0.05 considered statistically significant. The analysis of continuous variables was determined by data distribution and study design. For independent groups, normally distributed data were analyzed using independent samples t-tests, while non-normally distributed data were analyzed with the Wilcoxon rank-sum test (equivalent to the Mann–Whitney U test). For paired samples, the Wilcoxon signed-rank test was employed as the non-parametric alternative. Categorical variables (e.g., gender, smoking status, and alcohol consumption) were analyzed by chi-square tests or Fisher’s exact test between lung cancer patients and healthy controls.

## 3. Results

### 3.1. Basic Characteristics of the Enrolled Subjects

This study included a discovery cohort and two validation cohorts, encompassing 136 lung cancer patients and 199 healthy controls. Within the discovery cohort, we enrolled 24 patients diagnosed with stage IA-IVB lung cancer (LC) and 38 healthy control (HC) subjects. A total of 48 mouthwash samples were collected from the lung cancer patients (pre- and post-radiotherapy), alongside 38 samples obtained from the healthy control group. All patients underwent volumetric modulated arc therapy, receiving a total radiation dose ranging from 50.0 to 60.2 Gy within one month. Concurrent chemotherapy with paclitaxel and platinum-based drugs was administered to a subset of patients (n = 8, 33.3%). Patients with confirmed EGFR gene mutations received epidermal growth factor receptor tyrosine kinase inhibitors (EGFR-TKIs) (n = 4, 16.7%). Based on a 24-month PFS threshold, patients were categorized as responders (n = 14) or non-responders (n = 10). Detailed characteristics of all participants and the patients in the discovery cohort are presented in Table 1 and Table S2, respectively.

The two validation cohorts comprised publicly available 16S rRNA sequencing data from 112 patients and 161 healthy subjects. Detailed characteristics of the patients in the discovery and validation cohorts are presented in Table S3.

### 3.2. Baseline Oral Microbiota in Lung Cancer Patients Versus Matched Healthy Controls

**Figure 1:**
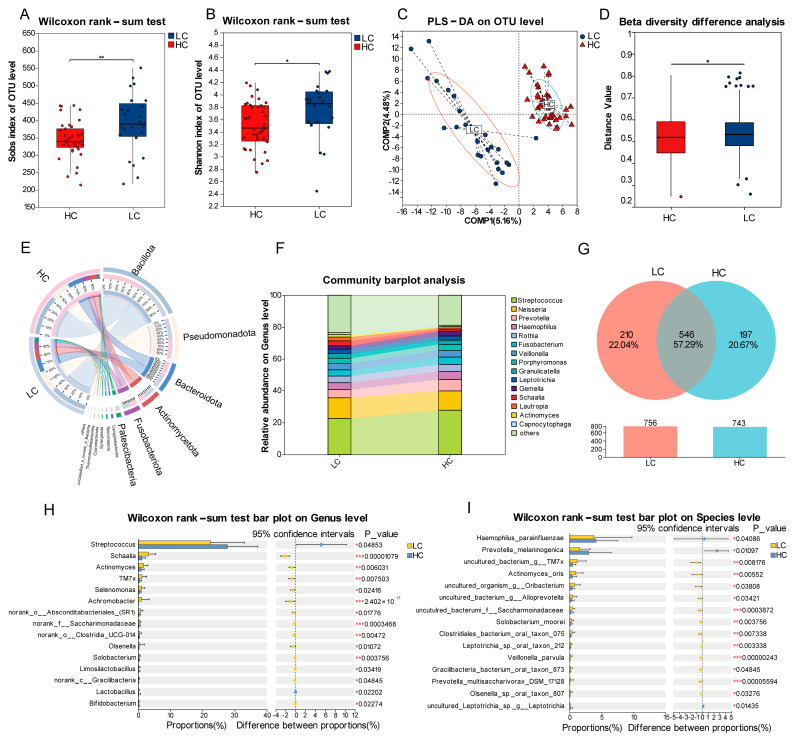
Comparative analysis of oral microbiota in baseline lung cancer patients and healthy controls. (**A**) Sobs index of OTU-level species between LC and HC groups. (**B**) Shannon index of OTU-level diversity difference. (**C**) Partial least squares discriminant analysis (PLS-DA) displays the distribution of LC and HC samples. (**D**) Beta diversity differences assessed through Wilcoxon rank-sum test. (**E**) Circos diagram shows relative abundance of Phylum Composition. (**F**) Genus composition between LC and HC groups. (**G**) Venn diagram of shared/unique operational taxonomic units (OTUs). (**H**,**I**) Differential Genera and Species: Bar plot showing abundance differences with 95% confidence interval (CI). Statistical significance is indicated as * *p* < 0.05, ** *p* < 0.01, *** *p* < 0.001.

To comprehensively characterize the oral microbial communities in LC patients, we compared microbiota profiles between LC patients at baseline and HC subjects. Alpha diversity analysis revealed significantly higher microbial richness (Sobs index) and diversity (Shannon index) in LC patients versus HC (Figure 1A,B, *p* = 0.010, *p* = 0.012). PLS-DA demonstrated distinct clustering between LC patients and HC subjects (Figure 1C), and significantly greater beta diversity dispersion was observed in LC patients compared with HC, as measured by Bray–Curtis distances (Figure 1D, *p* = 0.014).

At the phylum level, the dominant bacterial taxa included Bacillota, Pseudomonadota, Bacteroidota, Actinomycetota, and Fusobacteriota (Figure 1E). The predominant genera were *Streptococcus*, *Neisseria*, *Prevotella*, *Haemophilus*, and *Rothia* (Figure 1F). OTU analysis identified 546 shared OTUs (57.29%) between LC and HC groups, with 210 unique OTUs (22.04%) in the LC group and 197 unique OTUs (20.67%) in the HC group (Figure 1G).

Wilcoxon rank-sum tests were performed to identify differentially abundant taxa between LC and HC groups. *Streptococcus* (*p* = 0.049), *Haemophilus parainfluenzae* (*p* = 0.041), and *Prevotella melaninogenica* (*p* = 0.011) were enriched in the HC group. Conversely, the baseline LC group exhibited enrichment of multiple taxa, including genera *Schaalia* (*p* < 0.001), *Actinomyces* (*p* = 0.006), *TM7x* (*p* = 0.008), *Selenomonas* (*p* = 0.024), and *Achromobacter* (*p* < 0.001), along with species *Actinomyces oris* (*p* = 0.006), *Solobacterium moorei* (*p* = 0.004), and *Veillonella parvula* (*p* < 0.001) (Figure 1H,I).

### 3.3. Longitudinal Variation of Oral Microbiome Profiles Pre- and Post-Radiotherapy

**Figure 2:**
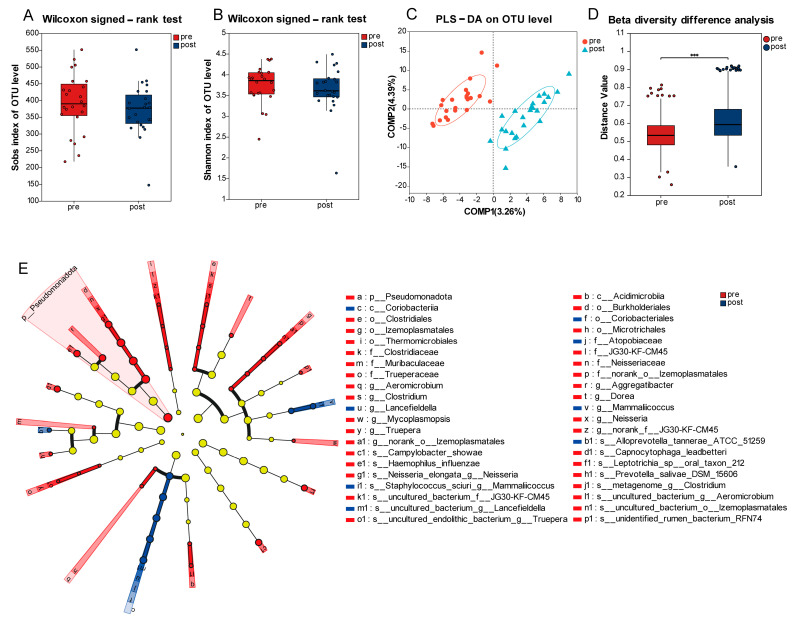
Oral microbiota dynamics in lung cancer patients before and after radiotherapy. (**A**) Sobs index shows richness change throughout the treatment. (**B**) Shannon index indicates diversity change. (**C**) PLS-DA reveals distinct group clustering. (**D**) Beta diversity differs between pre- and post-radiotherapy groups. (**E**) Cladogram illustrates differential microbial taxa determined by Linear Discriminant Analysis Effect Size (LEfSe) analysis (LDA score > 2). Statistical significance is indicated as *** *p* < 0.001.

Both the richness and diversity of the oral microbiota decreased after radiotherapy (Figure 2A,B). However, these decreases were not statistically significant (*p* = 0.153, *p* = 0.466, respectively), potentially attributable to the limited sample size and considerable inter-individual heterogeneity. PLS-DA revealed a significant shift in the composition and structure of the oral microbial communities from baseline to the end of radiotherapy treatment (Figure 2C). Furthermore, comparative analysis using Bray–Curtis distance demonstrated a significant difference in beta diversity between pre- and post-radiotherapy samples (Figure 2D, *p* < 0.001). To further elucidate the longitudinal dynamics of the oral microbiota throughout the treatment course, LEfSe analysis was performed. The results indicated significant enrichment of several taxa prior to radiotherapy, primarily including the orders Burkholderiales (*p* = 0.032) (family: Neisseriaceae, genus: *Neisseria*), Clostridiales (*p* = 0.009) (family: Clostridiaceae, genus: *clostridium*), Thermomicrobiales (*p* = 0.020), and Izemoplasmatales (*p* = 0.027), along with taxa at lower taxonomic levels. Conversely, the order Coriobacteriales (*p* = 0.035) and its subordinate taxa were significantly enriched after radiotherapy. At the genus level, the relative abundances of *Neisseria* (*p* = 0.019), *Aggregatibacter* (*p* = 0.049), and *Dorea* (*p* = 0.048) decreased following treatment (Figure 2E).

### 3.4. Identification of Microbial Taxa Associated with Treatment Response

**Figure 3:**
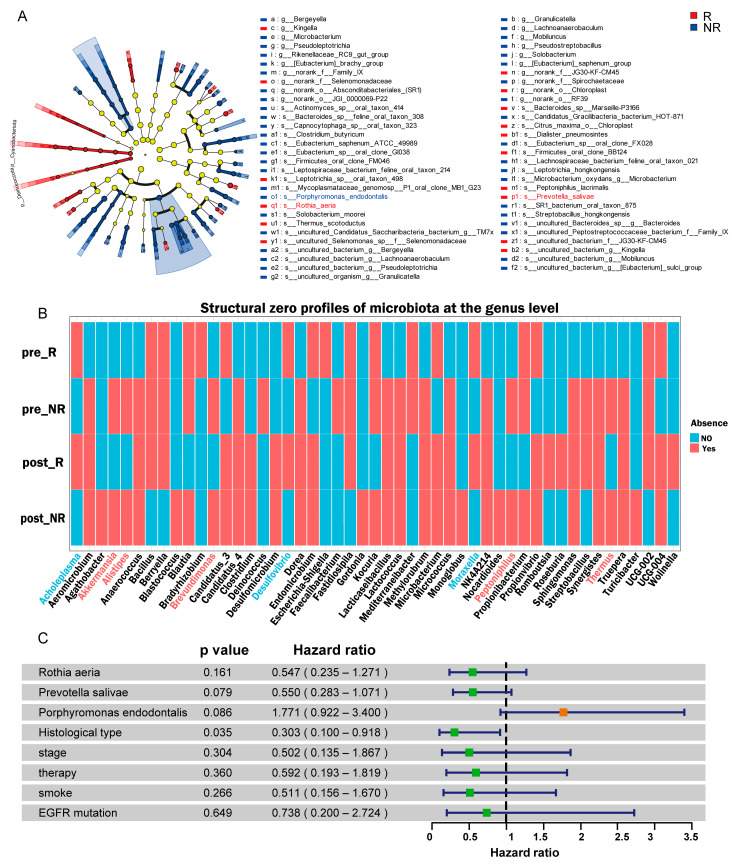
Identification of microbial taxa associated with treatment response. (**A**) The cladogram depicts significant microbial differences identified through LEfSe analysis between responders (R) and non-responders (NR) groups (LDA score > 2). (**B**) Heatmap showing the presence and absence of microbial genera across four groups identified through Analysis of Compositions of Microbiomes with Bias Correction 2 (ANCOM-BC2). The bacterial genera marked in red were virtually absent in non-responders, while those in blue were virtually absent in responders. (**C**) Forest plot of univariate Cox regression analysis assessing the association of oral microbial species and clinical factors with progression-free survival (PFS). Green symbols indicate the specific HR values for factors with HR < 1, and orange symbols represent factors with HR > 1. Error bars denote 95% confidence intervals.

To comprehensively identify microbiota associated with treatment response, we employed an integrated analytical approach utilizing the Wilcoxon rank-sum test, LEfSe and ANCOM-BC2 to detect differentially abundant taxa between R and NR groups. Wilcoxon rank-sum test revealed that responders exhibited higher abundances of *Prevotella salivae* and *Rothia aeria* (*p* = 0.014, *p* = 0.020), while *Porphyromonas endodontalis* predominated in non-responders (Figure S1A, *p* = 0.040). The differential abundance of these three taxa was further confirmed by the LEfSe analysis, which showed fully congruent enrichment trends: *Prevotella salivae* (LDA score: 3.29, *p* = 0.043) and *Rothia aeria* (LDA score: 3.75, *p* = 0.027) were enriched in responders, while *Porphyromonas endodontalis* (LDA score: 3.99, *p* = 0.037) was enriched in the non-responders (Figure 3A and Figure S1B). Consequently, these three taxa exhibited high LDA effect sizes, all notably above the conventional threshold of 2.0, underscoring their substantial biological relevance and strength as biomarkers for treatment response (Figure S1B).

To mitigate potential confounding by sampling time, we identified treatment response associated microbes by stratifying samples into four groups: pre_R, pre_NR, post_R, and post_NR. ANCOM-BC2 analysis indicated that *Akkermansia*, *Alistipes*, *Brevundimonas*, *Peptoniphilus* and *Thermus* were virtually absent in non-responders, whereas *Acholeplasma*, *Desulfovibrio* and *Moraxella* were virtually absent in responders (Figure 3B).

To assess the independent prognostic value of the microbial taxa, we performed a two-step Cox regression analysis. Univariate analysis indicated a consistent directional trend (Figure 3C): *Rothia aeria* (HR = 0.547, 95% CI: 0.235–1.271, *p* = 0.161) and *Prevotella salivae* (HR = 0.550, 95% CI: 0.283–1.071, *p* = 0.079) were associated with a reduced risk, while *Porphyromonas endodontalis* (HR = 1.771, 95% CI: 0.922–3.400, *p* = 0.086) was associated with an increased risk of progression. Histology (NSCLC vs. SCLC) was identified as a significant and strong prognostic factor (HR = 0.303, 95% CI: 0.100–0.918, *p* = 0.035). Therefore, to evaluate the independent contribution of the microbiome, we adjusted for histology in subsequent multivariate models.

Given the limited sample size, we included only histology along with one microbial variable per multivariate model to isolate their independent effects. After adjusting for histology, the association for *Rothia aeria* became statistically significant (HR = 0.470, 95% CI: 0.224–0.987, *p* = 0.046), as did that for *Porphyromonas endodontalis* (HR = 2.326, 95% CI: 1.054–5.135, *p* = 0.037). The protective trend for *Prevotella salivae* was also strengthened (HR = 0.528, 95% CI: 0.253–1.103, *p* = 0.089). This approach confirmed that accounting for the major clinical variable of histology was essential to revealing the independent relationship between these microbial biomarkers and PFS.

### 3.5. Negative Correlation of Oral Microbiota Richness and Diversity with ECOG Score and ProGRP Levels

**Figure 4:**
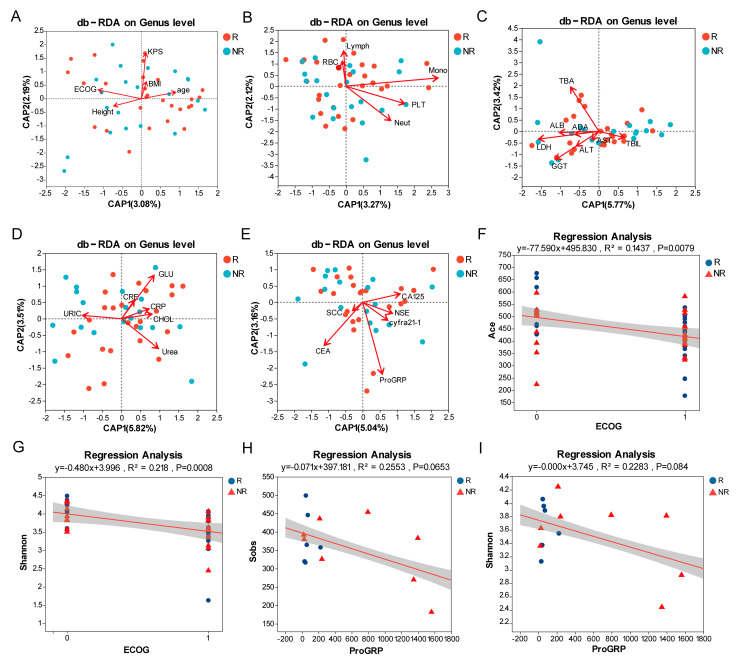
Analysis of microbial community structure and associations with clinical parameters. (**A**–**E**) db-RDA analysis shows the associations between clinical indices ((**A**): basic information; (**B**): complete blood count; (**C**,**D**): biochemical indicators; (**E**): tumor markers) and microbial community structure in responders and non-responders. (**F**–**I**) Regression analysis demonstrating the relationships between microbial alpha diversity indices (Ace, Sobs and Shannon index) and clinical parameters (Eastern Cooperative Oncology Group (ECOG) and pro-gastrin-releasing peptide (ProGRP)). R^2^ represents the proportion of variation explained by the regression line.

Through VIF analysis, we removed variables exhibiting multicollinearity, including weight, WBC, Hb, Eos, and Baso. To investigate the influence of the collected clinical indicators on the structure of the oral bacterial community, db-RDA was performed. Scatter plots illustrate the distribution of samples along principal components, and the direction and length of arrows allow interpretation of which clinical indicators significantly influence the microbial community structure. Our analysis revealed significant influences from several categories of variables. Among the basic information, ECOG score and KPS score significantly influenced microbial community structure (Figure 4A, *p* = 0.032, *p* = 0.001, respectively). Within the complete blood count parameters, monocyte, neutrophil and platelet showed significant influence (Figure 4B, *p* = 0.001, *p* = 0.006, *p* = 0.003, respectively). Regarding biochemical indicators, LDH, GGT, TBA, Urea, and GLU significantly affected the community structure (Figure 4C,D, *p* = 0.011, *p* = 0.004, *p* = 0.002, *p* = 0.039, *p* = 0.01, respectively). Finally, among the tumor markers, CEA and ProGRP were significant influencers (Figure 4E, *p* = 0.004, *p* = 0.001, respectively). These results collectively indicate the existence of complex regulatory mechanisms and networks between the microbiota and the host.

Subsequently, having identified these key clinical indicators impacting microbial community structure via Distance-based redundancy analysis (db-RDA), we further explored their correlations with microbial alpha diversity indices using regression analysis. The coefficient of determination (R^2^) indicates explanatory power of the clinical factor for differences in alpha diversity indices. We demonstrated that oral microbiota richness and diversity were negatively correlated with ECOG score through regression analysis (Figure 4F, R^2^ = 0.144, *p* = 0.008), (Figure 4G, R^2^ = 0.218, *p* < 0.001). Additionally, in SCLC patients, both oral microbiota richness (Figure 4H, R^2^ = 0.255, *p* = 0.065) and diversity (Figure 4I, R^2^ = 0.228, *p* = 0.084) were negatively correlated with ProGRP levels, each showing a trend towards significance. The modest R^2^ value reflects considerable residual variation, indicating limited explanatory power of the linear model. Unaccounted confounding variables or nonlinear relationships may contribute to this unexplained variance.

### 3.6. Construction of Lung Cancer Diagnosis Model and Treatment Response Prediction Model

**Figure 5:**
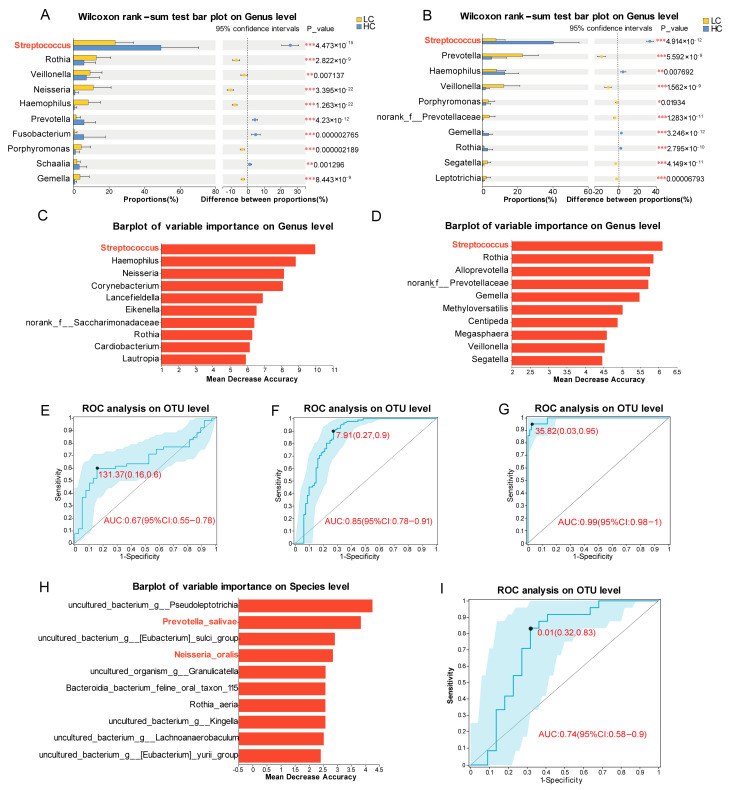
Construction of lung cancer diagnosis model and treatment response prediction model. (**A**,**B**) Wilcoxon rank-sum test bar plot on the genus level for lung cancer patients versus healthy controls in validation cohort 1 (**A**) and validation cohort 2 (**B**). (**C**,**D**) Genus-level importance ranking barplots derived from validation cohort 1 (**C**) and validation cohort 2 (**D**) for differentiating HC and LC groups. The x-axis represents mean decrease accuracy, quantifying each feature’s contribution to model prediction; the y-axis displays the top 10 genera ranked by importance. (**E**–**G**) Receiver operating characteristic (ROC) curve for the *Streptococcus*-based diagnostic model in the discovery cohort (**E**), validation cohort 1 (**F**) and validation cohort 2 (**G**), with area under the curve (AUC) indicating model performance. (**H**) Species importance ranking barplots derived from discovery cohort for differentiating response groups. The red fonts indicate the top two ranked and precisely annotated species. (**I**) ROC curve for the treatment response prediction model in the discovery cohort. Statistical significance is indicated as * *p* < 0.05, ** *p* < 0.01, *** *p* < 0.001.

To investigate the potential of oral microbiota as diagnostic biomarkers for lung cancer, we performed systematic microbial analyses across discovery and validation cohorts. Differential abundance analysis between healthy controls and LC patients in both the discovery and validation cohorts revealed that among the top 10 most abundant genera, only *Streptococcus* exhibited a conserved pattern of significantly higher abundance in HC groups across all datasets (Figure 1H and Figure 5A,B, *p* = 0.049, *p* < 0.001, *p* < 0.001, respectively). We subsequently applied random forest analysis to two independent validation cohorts and consistently identified *Streptococcus* as a top discriminatory taxon (Figure 5C,D). Building upon this finding, we developed a lung cancer diagnosis model based on *Streptococcus* abundance, which demonstrated robust diagnostic performance with area under the curve (AUC) values of 0.67 (discovery cohort, 95% CI: 0.55–0.78), 0.85 (validation cohort 1, 95% CI: 0.78–0.91), and 0.99 (validation cohort 2, 95% CI: 0.98–1) in ROC curve analysis (Figure 5E–G). The consistent abundance patterns of *Streptococcus* across diverse sample sets and the excellent diagnostic performance in validation cohorts strongly support the potential utility of oral *Streptococcus* as a non-invasive biomarker for lung cancer diagnosis.

To further assess the potential of oral microbiota as predictive biomarkers for lung cancer treatment response, random forest analysis was employed to identify and rank species distinguishing responders from non-responders (Figure 5H). A predictive model constructed using the top two ranked and precisely annotated species (*Prevotella salivae* and *Neisseria oralis*) achieved an AUC of 0.74 (95% CI: 0.58–0.90) for differentiating response groups (Figure 5I).

### 3.7. Association of Species-Level Microbiota with OS and PFS

To elucidate the prognostic potential of the treatment response associated microorganisms identified above, we further investigated the correlation between these species-level microbes and two critical clinical outcomes: OS and PFS. Optimal cutoff analysis was performed using the survminer package based on survival time and status. The optimal cutoff values for relative abundance were determined as follows: *Rothia aeria* (0.56%), *Prevotella salivae* (0.02%), and *Porphyromonas endodontalis* (0.27%). Samples with relative abundance above these thresholds were classified into the high group, while those below were assigned to the low group.

**Figure 6:**
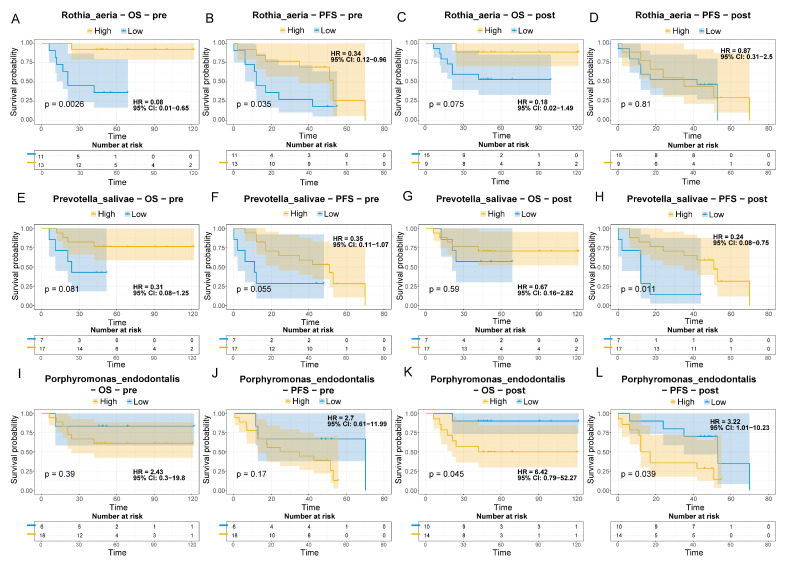
Survival analysis of lung cancer patients based on microbial markers. (**A**–**L**) Kaplan–Meier survival curves for Overall survival (OS) and PFS, stratified by high and low abundance of specific microbial species: *Rothia aeria* (**A**–**D**), *Prevotella salivae* (**E**–**H**), *Porphyromonas endoodontalis* (**I**–**L**). Log-rank tests were used to assess statistical significance, with p values indicated.

At baseline, a higher abundance of *Rothia aeria* was significantly associated with improved survival, corresponding to longer OS (HR = 0.08, 95% CI: 0.01–0.65, *p* = 0.0026, Figure 6A) and PFS (HR = 0.34, 95% CI: 0.12–0.96, *p* = 0.035, Figure 6B). In contrast, its abundance after treatment showed no significant association with either OS (*p* = 0.075, Figure 6C) or PFS (*p* = 0.81, Figure 6D).

For *Prevotella salivae*, higher baseline abundance showed only a non-significant trend toward better OS (*p* = 0.081, Figure 6E) and PFS (*p* = 0.055, Figure 6F). After treatment, however, higher abundance of *Prevotella salivae* was significantly associated with prolonged PFS (HR = 0.24, 95% CI: 0.08–0.75, *p* = 0.011, Figure 6H) but not with OS (*p* = 0.59, Figure 6G).

In the case of *Porphyromonas endodontalis*, lower baseline abundance exhibited a non-significant trend toward extended OS (*p* = 0.39, Figure 6I) and PFS (*p* = 0.17, Figure 6J). After treatment, lower abundance of *Porphyromonas endodontalis* was significantly associated with improved OS (HR = 6.42, 95% CI: 0.79–52.27, *p* = 0.045, Figure 6K) and PFS (HR = 3.22, 95% CI: 1.01–10.23, *p* = 0.039, Figure 6L).

Taken together, these results suggest that baseline levels of certain oral microbiota (e.g., *Rothia aeria*) may serve as favorable indicators, whereas the post-treatment state of others (e.g., *Porphyromonas endodontalis*) potentially functions as a biomarker for adverse outcomes. We note, however, that the present cohort is limited in size (n = 24), and the observed associations varied across taxa, time points, and endpoints. These findings thus offer preliminary, hypothesis-generating evidence to motivate future validation of time-specific microbial biomarkers in larger, prospective studies.

### 3.8. PICRUSt2 Predicts the Functions of Microbial Communities

**Figure 7:**
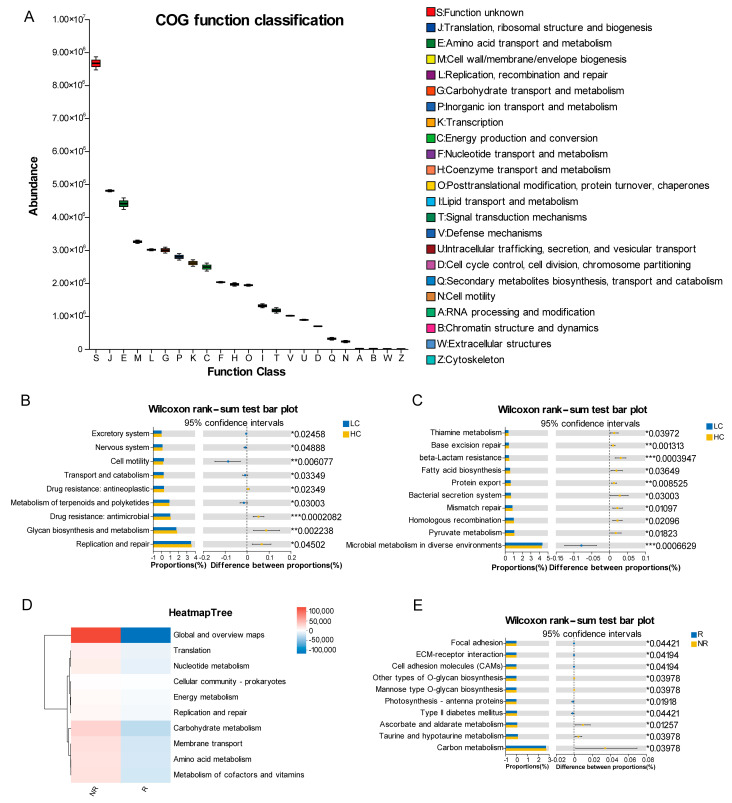
Functional profiling of microbial communities predicted by Phylogenetic investigation of communities by reconstruction of unobserved states 2 (PICRUSt2). (**A**) Abundance of Clusters of Orthologous Groups (COG) functional categories. (**B**,**C**) Proportional differences in Kyoto Encyclopedia of Genes and Genomes (KEGG) pathway level 2 (**B**) and level 3 (**C**) between LC and HC groups. (**D**,**E**) Proportional differences in KEGG pathway level 2 (**D**) and level 3 (**E**) between R and NR groups. Statistical significance is indicated as * *p* < 0.05, ** *p* < 0.01, *** *p* < 0.001.

Following characterization of the microbial community composition, we employed PICRUSt2 to predict the functional profiles of the microbiota in mouthwash samples, aiming to delineate microbial functional composition and abundance. Analysis using Clusters of Orthologous Groups (COG) functional classification revealed that microbial functions were primarily associated with translation, ribosomal structure and biogenesis; amino acid transport and metabolism; cell wall/membrane/envelope biogenesis; replication, recombination and repair; carbohydrate transport and metabolism (Figure 7A).

Subsequently, PICRUSt2 was utilized to identify differentially abundant KEGG pathways during lung carcinogenesis. At KEGG (Kyoto Encyclopedia of Genes and Genomes) pathway level 2, significant differences were primarily observed between lung cancer patients and healthy controls in replication and repair (*p* = 0.045), glycan biosynthesis and metabolism (*p* = 0.002), and drug resistance: antimicrobial (*p* < 0.001) (Figure 7B). At KEGG pathway level 3, microbial metabolism in diverse environments was significantly enriched in the lung cancer group (*p* < 0.001), whereas pyruvate metabolism (*p* = 0.018), homologous recombination (*p* = 0.021), and mismatch repair (*p* = 0.011) were significantly enriched in healthy controls (Figure 7C).

Furthermore, differential KEGG pathway analysis between responder and non-responder groups was conducted using PICRUSt2. At KEGG pathway level 2, pathways for metabolism of cofactors and vitamins, amino acid metabolism, and carbohydrate metabolism exhibited significant activation in the non-responders compared with responders (Figure 7D). At KEGG pathway level 3, carbon metabolism (*p* = 0.040), taurine and hypotaurine metabolism (*p* = 0.040), and ascorbate and aldarate metabolism (*p* = 0.013) were significantly activated in non-responders (Figure 7E).

## 4. Discussion

This study reveals the potential of the oral microbiome as a biomarker for lung cancer diagnosis and treatment monitoring across three independent cohorts. Radiotherapy induced significant alterations in the microbial community structure. Responders exhibited oral enrichment of *Rothia aeria* and *Prevotella salivae*, which correlated with improved OS and PFS. Conversely, non-responders harbored a higher abundance of *Porphyromonas endodontalis*, associated with poorer prognosis. The *Streptococcus*-based diagnostic model demonstrated excellent discriminative power in two independent validation cohorts, achieving AUCs of 0.85 and 0.99. Additionally, the therapeutic response prediction model utilizing *Prevotella salivae* and *Neisseria oralis* yielded an AUC of 0.74 for distinguishing responders from non-responders. Collectively, these findings indicate that the oral microbiome holds promise as a non-invasive biomarker for lung cancer diagnosis and treatment efficacy prediction.

This study reveals significant differences in the composition of the oral microbial community between lung cancer patients and healthy controls. Notably, at the species level, the HC group exhibited a higher abundance of *Haemophilus parainfluenzae* and *Prevotella melaninogenica*, whereas the baseline LC group showed elevated levels of *Actinomyces oris* and *Solobacterium moorei*. *Haemophilus parainfluenzae* typically colonizes distinct niches in the human nasopharynx and periodontal mucosa, and the genus *Haemophilus* constitutes approximately 10% of the upper respiratory tract’s bacterial flora [17]. Interestingly, studies have reported relatively higher salivary levels of *H. parainfluenzae* in controls compared with pancreatic ductal adenocarcinoma (PDAC) patients [18]. Furthermore, its oral abundance has been negatively correlated with salivary chemokines CCL13, CCL24, CXCL11, and CXCL13, which are known mediators of periodontal tissue destruction [19]. Consequently, *H. parainfluenzae* is postulated to exert a protective role for the host, in line with our observations. In addition, Mager et al. reported increased *Prevotella melaninogenica* in the saliva of oral cancer patients, suggesting its potential as a diagnostic biomarker for oral squamous cell carcinoma [20]. Our findings indicate greater abundance in healthy controls, and this discrepancy may be attributable to differences in tumor type. Concerning LC-enriched species, *Actinomyces oris* abundance was reported to be 6.5-fold higher in the feces of Parkinson’s disease patients versus controls [21]. We similarly observed higher oral abundance of *Actinomyces oris* in mouthwash samples of LC patients, although its specific role and mechanism in tumorigenesis remain unclear. Furthermore, analysis of fecal microbiota revealed significantly higher levels of *Solobacterium moorei* in both intramucosal carcinoma and colorectal cancer (CRC) patients compared with healthy controls, with the highest abundance in intramucosal carcinoma. This suggests a potential involvement of *S. moorei* in early colorectal carcinogenesis [22]. Our finding of elevated *S. moorei* in mouthwash samples from LC patients aligns with this, indicating it may play a role in the early progression of diverse tumor types.

Shifting focus to treatment response, our study identified differential microbial taxa between responders and non-responders. ANCOM-BC2 analysis indicated that *Akkermansia* was nearly absent in non-responders. The genus *Akkermansia* comprises at least eight resident gastrointestinal species [23]. Research predominantly focuses on *Akkermansia muciniphila*, which accounts for 1–4% of the adult gut microbiota [24]. Supporting a beneficial role, a prospective multicenter study linked increased fecal *A. muciniphila* to significant clinical benefit from immune checkpoint inhibitors in advanced NSCLC patients, manifesting as improved objective response rate and OS [25]. Mechanistically, oral *A. muciniphila* supplementation restored PD-1 blockade efficacy in mice by enhancing recruitment of CCR9^+^CXCR3^+^CD4^+^ T lymphocytes to tumors [26]. Additionally, *Akkermansia* enhanced cisplatin’s anti-tumor effects in a Lewis lung cancer model [27]. Our observation of significantly higher oral *Akkermansia* levels in responders compared with non-responders is thus consistent with the reported beneficial functions of this genus. Conversely, ANCOM-BC2 analysis also suggested the near absence of *Desulfovibrio* and *Moraxella* in responders. This is notable because *Desulfovibrio* produces leucine, which promotes expansion of myeloid-derived suppressor cells, accelerating breast cancer growth [28]. Moreover, *Desulfovibrio vulgaris* flagellin exacerbates colorectal cancer epithelial–mesenchymal transition via LRRC19/TRAF6/TAK1 signaling pathway activation, promoting tumor growth [29]. Similarly, *Moraxella* was found enriched in lower respiratory tract samples from stage IIIB-IV NSCLC patients versus those with stage I-IIIA disease [30]. Collectively, these findings suggest that *Desulfovibrio* and *Moraxella* may contribute to tumor initiation or progression.

This study further explored the influence of clinical factors on oral bacterial community structure, identifying ECOG performance status and ProGRP levels as significant determinants. ECOG status is a critical prognostic parameter that guides therapeutic decisions in oncology, whereas ProGRP serves as a sensitive biomarker for diagnosing SCLC, monitoring treatment response, and detecting early relapse. ProGRP is notably stable in peripheral blood, and levels > 150 pg/mL confer a 93.7% probability of SCLC [31]. Nevertheless, ECOG status or ProGRP accounted for less than 30% of the variance in oral α diversity indices, indicating that additional clinical or environmental factors exert substantial influence. Consistent with prior reports, higher oral microbial α diversity has been inversely associated with lung cancer risk [32]. These observations underscore the need for comprehensive investigations into the determinants of oral microbiome diversity.

Using random-forest modelling, we next constructed models for lung cancer diagnosis and treatment response prediction. A *Streptococcus*-based classifier achieved an AUC of 0.67 in the discovery cohort, likely attributable to several factors. First, the limited sample size (n = 62) may have reduced statistical power. Second, significant clinical heterogeneity was present, including: (i) mixed histopathological subtypes (both NSCLC and SCLC); (ii) varying tumor stages; (iii) heterogeneous treatment regimens ranging from definitive chemoradiation to molecularly targeted therapy combinations. These clinical variabilities potentially introduced substantial confounding effects that may have compromised model accuracy. Encouragingly, the model demonstrated strong discriminatory capacity in two independent validation sets (AUC = 0.85 and 0.99), supporting the clinical relevance of oral *Streptococcus* for lung cancer detection. *Streptococcus* is among the most abundant genera in both the oral cavity and the lower respiratory tract, encompassing species such as *S. anginosus*, *S. mutans*, and *S. salivarius* [33]. Its relative abundance varies markedly across anatomical sites and different samples, implying potentially divergent functional roles [34]. Epidemiological data indicate a positive association between oral *Streptococcus* abundance and lung cancer risk, particularly in squamous cell carcinoma patients and former smokers [32]. Studies further reveal that oral-derived *Streptococcus* is enriched in lung cancer airways, where it is linked to activation of ERK and PI3K signalling—pathways implicated in tumorigenesis [35]. In addition, *S. anginosus* promotes gastric tumorigenesis by directly interacting with gastric epithelial cells on the TMPC-ANXA2-MAPK axis [36]. Conversely, a higher abundance of gut *Streptococcus* has been reported in healthy controls compared with lung cancer patients [37], mirroring our observation of elevated *Streptococcus* levels in the oral microbiome from healthy controls. Collectively, these data highlight the genus-wide heterogeneity of *Streptococcus* and the necessity for species-level resolution to delineate its oncogenic or protective roles. Additionally, we constructed a therapeutic response prediction model based on the abundance of *Prevotella salivae* and *Neisseria oralis*. The absence of publicly available 16S rRNA datasets annotated with treatment response metadata precluded external validation; nevertheless, our findings provide novel insights into oral microbial signature associated with therapy response in lung cancer. Supporting the role of *Prevotella salivae*, prospective studies involving three large cohorts revealed an association between salivary *Prevotella salivae* and a reduced risk of head and neck squamous cell carcinoma, consistent with our findings [38]. However, the precise mechanisms by which *P. salivae* improves cancer patient prognosis remain unclear. Regarding *Neisseria oralis*, high oral *N. oralis* abundance has been linked to accelerated progression of colorectal cancer [39], whereas our data reveal a positive association between *N. oralis* and overall survival in lung cancer patients. These divergent outcomes suggest that *N. oralis* exerts tumour-type-specific effects, likely mediated by distinct microenvironmental interactions.

Our study identified three oral bacterial species significantly associated with lung cancer prognosis: *Rothia aeria* and *Prevotella salivae* emerged as non-invasive biomarkers indicative of favorable prognosis, while *Porphyromonas endodontalis* served as a biomarker for poor prognosis. Supporting the protective role of *Rothia aeria*, a study in periodontitis patients revealed a positive correlation between its abundance and salivary CCL2 levels; CCL2 mitigates inflammation by reducing the M1:M2 macrophage ratio in gingival tissue and prevents alveolar bone loss in murine models [19]. Our findings also reveal that oral *R. aeria* exerts a beneficial effect in lung cancer, although its precise mechanistic role in improving cancer outcomes remains undefined. Conversely, we observed significantly increased oral *Porphyromonas endodontalis* in non-responders. *P. endodontalis*, a Gram-negative anaerobe, is a causative agent of acute periapical lesions in infected root canals [40]. Importantly, this finding resonates with broader oncological evidence. First, salivary levels of *P. endodontalis* are significantly elevated in patients with early-stage intramucosal esophageal squamous cell carcinoma relative to healthy controls and have been proposed as a diagnostic biomarker [41]. Second, the organism is enriched in the saliva of oral squamous cell carcinoma patients, where it may foster tumorigenesis through MMP-13 up-regulation [42]. Third, gastric lavage fluid from advanced gastric adenocarcinoma patients contains higher *P. endodontalis* loads than samples from superficial gastritis controls [43]. Finally, *P. gingivalis* is established as a key driver in CRC and PDAC progressio n [44,45]. Taken together, these converging lines of evidence support a broader role for *P. endodontalis* in tumor initiation and progression across multiple malignancies.

The PICRUSt2 analysis revealed functional differences in the oral microbiome associated with lung carcinogenesis and treatment response, providing new insights into potential underlying mechanisms. Notably, 16S rRNA-based functional prediction revealed that healthy controls exhibited higher enrichment of replication and repair pathways (KEGG level 2) compared to lung cancer patients, including homologous recombination and mismatch repair at KEGG level 3. This suggests that these pathways are crucial for maintaining homeostatic regulation. In healthy individuals, homologous recombination is crucial for maintaining genomic stability by accurately repairing DNA double-strand breaks. Compromised homologous recombination drives genomic instability and significantly elevates cancer risk [46].

Furthermore, functional disparities between treatment responders and non-responders were particularly pronounced. Non-responders exhibited activation of pathways related to cofactor and vitamin metabolism, amino acid metabolism, and carbohydrate metabolism. As shown in prior studies, inhibiting glycolysis or glutamine using specific inhibitors could radiosensitize certain NSCLC cell lines, with efficacy closely tied to molecular features such as KRAS mutation status [47]. The activation of amino acid and carbohydrate metabolism in non-responders may promote the synthesis of ATP, lipids, and amino acids required for proliferation, as well as enhance glutathione production to counteract oxidative stress [48]. The enrichment of taurine metabolism in non-responders is especially noteworthy. Microbially mediated taurine metabolism can influence host health by generating pro-inflammatory secondary bile acids and genotoxic hydrogen sulfide, potentially shaping a pro-tumor microenvironment [49]. Although these functional features remain predictive, they point to important directions for future research. Future studies employing metatranscriptomics are warranted to validate these functional predictions.

Collectively, our multi-cohort analysis supports the oral microbiome as a biomarker reservoir for lung cancer diagnosis and radiotherapy response monitoring. While this study provides valuable insights, several limitations should be considered: (1) Sample size and clinical heterogeneity: The discovery cohort’s modest sample size and clinical heterogeneity in tumor characteristics (including stage, histology) and treatment modalities may introduce confounding effects. This issue was further compounded in the comparison between responders and non-responders, which involved even smaller subgroups, thereby compromising the stability of the identified microbiota features and potentially inflating effect sizes. (2) Baseline differences across cohorts: Although we validated our findings in two independent cohorts, significant baseline differences persisted between discovery and validation sets. Notably, variations in smoking history, EGFR mutation status, tumor histopathology, and treatment regimens may influence the observed microbial profiles [50]. (3) Absence of parallel longitudinal sampling: Oral wash samples from healthy controls were collected at a single time point, whereas samples from lung cancer patients were obtained longitudinally, before and after radiotherapy. The absence of parallel longitudinal sampling in healthy individuals means we cannot fully account for potential natural temporal variation within the healthy oral microbiome. This may prevent direct comparison of temporal dynamics between the two groups. Future studies should incorporate multi-center cohorts with larger sample sizes to control the above confounding factors, utilizing 16S rRNA or metagenomic sequencing to precisely identify differentially abundant taxa and functional pathways, clarifying their underlying mechanisms.

## 5. Conclusions

Lung cancer patients harbor distinct and dynamic oral microbial signatures that significantly correlate with disease status, therapeutic response, and survival outcomes. Keystone commensal *Streptococcus* discriminates patients from healthy individuals, while responder-enriched taxa (*Rothia aeria*, *Prevotella salivae*) and non-responder-associated taxa (*Porphyromonas endodontalis*) predict radiotherapy efficacy and survival. Externally validated diagnostic model (AUCs: 0.85 and 0.99 in two cohorts) and response prediction model (AUC: 0.74) underscore the oral microbiome as a robust, non-invasive biomarker. The negative correlations observed between microbial diversity and clinical markers ECOG score, ProGRP level support integrating oral microbiota profiling into promising biomarkers to enhance risk stratification and outcome monitoring in lung cancer management.

## Figures and Tables

**Table 1:** Characteristics of the discovery cohort subjects.

Characteristic	Healthy Control (HC), (n = 38)	Lung Cancer (LC), (n = 24)	*p* Value
Age (years), Median (IQR)	53 (7)	61 (16)	0.025
Gender, n (%)			0.117
Female	17 (44.7)	6 (25.0)	
Male	21 (55.3)	18 (75.0)	
Smoking, n (%)			0.062
Nonsmoker	25 (65.8)	10 (41.7)	
Current or former smoker	13 (34.2)	14 (58.3)	
Alcohol, n (%)			0.704
Absent	24 (63.2)	14 (58.3)	
Present	14 (36.8)	10 (41.7)	
BMI (kg/m^2^), (Mean ± SD)	24.54 ± 3.54	24.00 ± 3.20	0.550

## Data Availability

The raw sequencing data reported in this paper have been deposited in the Genome Sequence Archive in the National Genomics Data Center, China National Center for Bioinformation/Beijing Institute of Genomics, Chinese Academy of Sciences (GSA: CRA034602) that are publicly accessible at https://ngdc.cncb.ac.cn/gsa.

## Supplementary Materials

The following supporting information can be downloaded at: https://www.mdpi.com/article/10.3390/pathogens14121294/s1, Figure S1: Wilcoxon rank-sum test and LDA effect size analysis of differential species between R and NR groups; Table S1: The source and profile of two validation cohorts; Table S2: Baseline characteristics of responders and non-responders in the discovery cohort; Table S3: Characteristics of patients in the discovery and validation cohorts.

## Abbreviations

The following abbreviations are used in this manuscript:

NSCLC	Non-small cell lung cancer
SCLC	Small cell lung cancer
ECOG	Eastern Cooperative Oncology Group
ProGRP	Pro-gastrin-releasing peptide
OTUs	Operational taxonomic units
PLS-DA	Partial least squares discriminant analysis
LEfSe	Linear Discriminant Analysis Effect Size
ANCOM-BC2	Analysis of Compositions of Microbiomes with Bias Correction 2
VIF	Variance inflation factors
db-RDA	Distance-based redundancy analysis
ROC	Receiver operating characteristic
OS	Overall survival
PFS	Progression-free survival
PICRUSt2	Phylogenetic investigation of communities by reconstruction of unobserved states 2
R	Responders
NR	Non-responders
LC	Lung cancer
HC	Healthy control
AUC	Area under the curve
EGFR-TKIs	Epidermal growth factor receptor tyrosine kinase inhibitors
COG	Clusters of Orthologous Groups
KEGG	Kyoto Encyclopedia of Genes and Genomes
PDAC	Pancreatic ductal adenocarcinoma
CRC	Colorectal cancer

## References

[1] Bray F., Laversanne M., Sung H., Ferlay J., Siegel R.L., Soerjomataram I., Jemal A. (2024). Global cancer statistics 2022: GLOBOCAN estimates of incidence and mortality worldwide for 36 cancers in 185 countries. CA Cancer J. Clin..

[2] Leiter A., Veluswamy R.R., Wisnivesky J.P. (2023). The global burden of lung cancer: Current status and future trends. Nat. Rev. Clin. Oncol..

[3] Kitamoto S., Nagao-Kitamoto H., Hein R., Schmidt T.M., Kamada N. (2020). The Bacterial Connection between the Oral Cavity and the Gut Diseases. J. Dent. Res..

[4] Huang D., Chen Y., Li C., Yang S., Lin L., Zhang X., Su X., Liu L., Zhao H., Luo T. (2025). Variations in salivary microbiome and metabolites are associated with immunotherapy efficacy in patients with advanced NSCLC. mSystems.

[5] Lim M.Y., Hong S., Hwang K.H., Lim E.J., Han J.Y., Nam Y.D. (2021). Diagnostic and prognostic potential of the oral and gut microbiome for lung adenocarcinoma. Clin. Transl. Med..

[6] Abola I., Gudra D., Ustinova M., Fridmanis D., Emulina D.E., Skadins I., Brinkmane A., Lauga-Tunina U., Gailite L., Auzenbaha M. (2023). Oral Microbiome Traits of Type 1 Diabetes and Phenylketonuria Patients in Latvia. Microorganisms.

[7] Sun Y., Liu Y., Li J., Tan Y., An T., Zhuo M., Pan Z., Ma M., Jia B., Zhang H. (2023). Characterization of Lung and Oral Microbiomes in Lung Cancer Patients Using Culturomics and 16S rRNA Gene Sequencing. Microbiol. Spectr..

[8] Liu S., Song Q., Zhang C., Li M., Li Z., Liu Y., Xu L., Xie X., Zhao L., Zhang R. (2023). Saliva microbiome alterations in dental fluorosis population. J. Oral Microbiol..

[9] Liu C., Zhao D., Ma W., Guo Y., Wang A., Wang Q., Lee D.-J. (2015). Denitrifying sulfide removal process on high-salinity wastewaters in the presence of *Halomonas* sp. Appl. Microbiol. Biotechnol..

[10] Chen S., Zhou Y., Chen Y., Gu J. (2018). fastp: An ultra-fast all-in-one FASTQ preprocessor. Bioinformatics.

[11] Magoč T., Salzberg S.L. (2011). FLASH: Fast length adjustment of short reads to improve genome assemblies. Bioinformatics.

[12] Edgar R.C. (2013). UPARSE: Highly accurate OTU sequences from microbial amplicon reads. Nat. Methods.

[13] Schloss P.D., Westcott S.L., Ryabin T., Hall J.R., Hartmann M., Hollister E.B., Lesniewski R.A., Oakley B.B., Parks D.H., Robinson C.J. (2009). Introducing mothur: Open-Source, Platform-Independent, Community-Supported Software for Describing and Comparing Microbial Communities. Appl. Environ. Microbiol..

[14] Segata N., Izard J., Waldron L., Gevers D., Miropolsky L., Garrett W.S., Huttenhower C. (2011). Metagenomic biomarker discovery and explanation. Genome Biol..

[15] Lin H., Peddada S.D. (2023). Multigroup analysis of compositions of microbiomes with covariate adjustments and repeated measures. Nat. Methods.

[16] Douglas G.M., Maffei V.J., Zaneveld J.R., Yurgel S.N., Brown J.R., Taylor C.M., Huttenhower C., Langille M.G.I. (2020). PICRUSt2 for prediction of metagenome functions. Nat. Biotechnol..

[17] Mukundan D., Ecevit Z., Patel M., Marrs C.F., Gilsdorf J.R. (2007). Pharyngeal Colonization Dynamics of *Haemophilus influenzae* and *Haemophilus haemolyticus* in Healthy Adult Carriers. J. Clin. Microbiol..

[18] Olson S.H., Satagopan J., Xu Y., Ling L., Leong S., Orlow I., Saldia A., Li P., Nunes P., Madonia V. (2017). The oral microbiota in patients with pancreatic cancer, patients with IPMNs, and controls: A pilot study. Cancer Causes Control.

[19] Kawamoto D., Borges R., Ribeiro R.A., de Souza R.F., Amado P.P.P., Saraiva L., Horliana A.C.R.T., Faveri M., Mayer M.P.A. (2021). Oral Dysbiosis in Severe Forms of Periodontitis Is Associated with Gut Dysbiosis and Correlated with Salivary Inflammatory Mediators: A Preliminary Study. Front. Oral Health.

[20] Mager D.L., Haffajee A.D., Devlin P.M., Norris C.M., Posner M.R., Goodson J.M. (2005). The salivary microbiota as a diagnostic indicator of oral cancer: A descriptive, non-randomized study of cancer-free and oral squamous cell carcinoma subjects. J. Transl. Med..

[21] Wallen Z.D., Demirkan A., Twa G., Cohen G., Dean M.N., Standaert D.G., Sampson T.R., Payami H. (2022). Metagenomics of Parkinson’s disease implicates the gut microbiome in multiple disease mechanisms. Nat. Commun..

[22] Yachida S., Mizutani S., Shiroma H., Shiba S., Nakajima T., Sakamoto T., Watanabe H., Masuda K., Nishimoto Y., Kubo M. (2019). Metagenomic and metabolomic analyses reveal distinct stage-specific phenotypes of the gut microbiota in colorectal cancer. Nat. Med..

[23] El-Sayed N., van Passel M.W.J., Kant R., Zoetendal E.G., Plugge C.M., Derrien M., Malfatti S.A., Chain P.S.G., Woyke T., Palva A. (2011). The Genome of Akkermansia muciniphila, a Dedicated Intestinal Mucin Degrader, and Its Use in Exploring Intestinal Metagenomes. PLoS ONE.

[24] Belzer C., de Vos W.M. (2012). Microbes inside—From diversity to function: The case of Akkermansia. ISME J..

[25] Derosa L., Routy B., Thomas A.M., Iebba V., Zalcman G., Friard S., Mazieres J., Audigier-Valette C., Moro-Sibilot D., Goldwasser F. (2022). Intestinal Akkermansia muciniphila predicts clinical response to PD-1 blockade in patients with advanced non-small-cell lung cancer. Nat. Med..

[26] Routy B., Le Chatelier E., Derosa L., Duong C.P.M., Alou M.T., Daillère R., Fluckiger A., Messaoudene M., Rauber C., Roberti M.P. (2018). Gut microbiome influences efficacy of PD-1-based immunotherapy against epithelial tumors. Science.

[27] Chen Z., Qian X., Chen S., Fu X., Ma G., Zhang A. (2020). Akkermansia muciniphila Enhances the Antitumor Effect of Cisplatin in Lewis Lung Cancer Mice. J. Immunol. Res..

[28] Chen J., Liu X., Zou Y., Gong J., Ge Z., Lin X., Zhang W., Huang H., Zhao J., Saw P.E. (2024). A high-fat diet promotes cancer progression by inducing gut microbiota–mediated leucine production and PMN-MDSC differentiation. Proc. Natl. Acad. Sci. USA.

[29] Dong Y., Meng F., Wang J., Wei J., Zhang K., Qin S., Li M., Wang F., Wang B., Liu T. (2024). Desulfovibrio vulgaris flagellin exacerbates colorectal cancer through activating LRRC19/TRAF6/TAK1 pathway. Gut Microbes.

[30] Tsay J.-C.J., Wu B.G., Sulaiman I., Gershner K., Schluger R., Li Y., Yie T.-A., Meyn P., Olsen E., Perez L. (2021). Lower Airway Dysbiosis Affects Lung Cancer Progression. Cancer Discov..

[31] Wojcik E., Kulpa J. (2017). Pro-gastrin-releasing peptide (ProGRP) as a biomarker in small-cell lung cancer diagnosis, monitoring and evaluation of treatment response. Lung Cancer Targets Ther..

[32] Vogtmann E., Hua X., Yu G., Purandare V., Hullings A.G., Shao D., Wan Y., Li S., Dagnall C.L., Jones K. (2022). The Oral Microbiome and Lung Cancer Risk: An Analysis of 3 Prospective Cohort Studies. J. Natl. Cancer Inst..

[33] Li R., Li J., Zhou X. (2024). Lung microbiome: New insights into the pathogenesis of respiratory diseases. Signal Transduct. Target. Ther..

[34] Lucaciu S.-R., Domokos B., Puiu R., Ruta V., Motoc S.N., Rajnoveanu R., Todea D., Stoia A.M., Man A.M. (2024). Lung Microbiome in Lung Cancer: A Systematic Review. Microorganisms.

[35] Tsay J.-C.J., Wu B.G., Badri M.H., Clemente J.C., Shen N., Meyn P., Li Y., Yie T.-A., Lhakhang T., Olsen E. (2018). Airway Microbiota Is Associated with Upregulation of the PI3K Pathway in Lung Cancer. Am. J. Respir. Crit. Care Med..

[36] Fu K., Cheung A.H.K., Wong C.C., Liu W., Zhou Y., Wang F., Huang P., Yuan K., Coker O.O., Pan Y. (2024). *Streptococcus anginosus* promotes gastric inflammation, atrophy, and tumorigenesis in mice. Cell.

[37] Zheng Y., Fang Z., Xue Y., Zhang J., Zhu J., Gao R., Yao S., Ye Y., Wang S., Lin C. (2020). Specific gut microbiome signature predicts the early-stage lung cancer. Gut Microbes.

[38] Kwak S., Wang C., Usyk M., Wu F., Freedman N.D., Huang W.-Y., McCullough M.L., Um C.Y., Shrubsole M.J., Cai Q. (2024). Oral Microbiome and Subsequent Risk of Head and Neck Squamous Cell Cancer. JAMA Oncol..

[39] Zhou S.-H., Du Y., Xue W.-Q., He M.-J., Zhou T., Zhao Z.-Y., Pei L., Chen Y.-W., Xie J.-R., Huang C.-L. (2025). Oral microbiota signature predicts the prognosis of colorectal carcinoma. npj Biofilms Microbiomes.

[40] Ahmed S.A., Nishimata H., Ohara-Nemoto Y., Baba T.T., Hoshino T., Fujiwara T., Shimoyama Y., Kimura S., Nemoto T.K. (2014). Identification of Dipeptidyl-Peptidase (DPP)5 and DPP7 in *Porphyromonas endodontalis*, Distinct from Those in *Porphyromonas gingivalis*. PLoS ONE.

[41] Chen H., Jiang X., Zhu F., Yang R., Yu X., Zhou X., Tang N. (2024). Characteristics of the oral and gastric microbiome in patients with early-stage intramucosal esophageal squamous cell carcinoma. BMC Microbiol..

[42] Rai A.K., Panda M., Das A.K., Rahman T., Das R., Das K., Sarma A., Kataki A.C., Chattopadhyay I. (2020). Dysbiosis of salivary microbiome and cytokines influence oral squamous cell carcinoma through inflammation. Arch. Microbiol..

[43] Hu Y.-L., Pang W., Huang Y., Zhang Y., Zhang C.-J. (2018). The Gastric Microbiome Is Perturbed in Advanced Gastric Adenocarcinoma Identified Through Shotgun Metagenomics. Front. Cell. Infect. Microbiol..

[44] Cai P., Xiong J., Sha H., Dai X., Lu J. (2023). Tumor bacterial markers diagnose the initiation and four stages of colorectal cancer. Front. Cell. Infect. Microbiol..

[45] Zhu Y., Liang X., Zhi M., Li L., Zhang G., Chen C., Wang L., Wang P., Zhong N., Feng Q. (2024). Succession of the multi-site microbiome along pancreatic ductal adenocarcinoma tumorigenesis. Front. Immunol..

[46] Jasin M., Rothstein R. (2013). Repair of strand breaks by homologous recombination. Cold Spring Harb. Perspect. Biol..

[47] Meijer T.W.H., Peeters W.J.M., Dubois L.J., van Gisbergen M.W., Biemans R., Venhuizen J.-H., Span P.N., Bussink J. (2018). Targeting glucose and glutamine metabolism combined with radiation therapy in non-small cell lung cancer. Lung Cancer.

[48] Lunt S.Y., Vander Heiden M.G. (2011). Aerobic Glycolysis: Meeting the Metabolic Requirements of Cell Proliferation. Annu. Rev. Cell Dev. Biol..

[49] Duszka K. (2022). Versatile Triad Alliance: Bile Acid, Taurine and Microbiota. Cells.

[50] Huang Q., Wu X., Zhou X., Sun Z., Shen J., Kong M., Chen N., Qiu J.-G., Jiang B.-H., Yuan C. (2023). Association of cigarette smoking with oral bacterial microbiota and cardiometabolic health in Chinese adults. BMC Microbiol..

