# Supplementary material for: Identification of Oral Microbiome Biomarkers Associated with Lung Cancer Diagnosis and Radiotherapy Response Prediction
Source: Pathogens. 2025 Dec 16;14(12):1294. doi: 10.3390/pathogens14121294 (PMC12735506; doi:10.3390/pathogens14121294)
Supplement: Supplementary file 1 [file pathogens-14-01294-s001.zip › Table S1-3.pdf]

Table S1. The source and profile of two validation cohorts

Cohorts	Sequencing platform and read length	Sequencing primers	Sample	Data
Validation cohort 1	Illumina MiSeq, 300-bp paired-end reads	341F(5'-TCGTCGGCAGCGTCAGAGTGTATAAGAGACAGCCTACCGGNGGCWGCAG-3') and 805R(5'-GTCTCGTGGGCTCGGAGATGTATAAGAGACAGGACTACHVGGGTATCTAATCC-3')	91 lung cancer patients	PRJEB44168
		341F(5'-TCGTCGGCAGCGTCAGATGTGTATAAGAGACAGNNNNNNCCTACGGGNGGCWGCAG-3') and 805R(5'-GTCTCGTGGGCTCGGAGATGTGTATAAGAGACAGNNNNNNGACTACHVGGGTATCTAATCC-3')	81 healthy controls	PRJEB48982

Validation cohort 2	Illumina NovaSeq 6000 , 250-bp paired-end reads	341F(5′-CCTAYGGGRBGCASCAG-3′) and 806R(5′-GGACTACNNGGGTATCTAAT-3′)	21 lung cancer patients	PRJNA904049
		341F(5′-CCTAYGGGRBGCASCAG-3′) and 806R(5′-GGACTACNNGGGTATCTAAT-3′)	80 healthy controls	PRJNA822496

Table S2. Baseline characteristics of responders and non-responders in the discovery cohort

	Responders, (n=14)	Non-responders, (n=10)	*P* value
Smoking, n (%)			0.104
Nonsmoker	8 (57.1)	2 (20.0)	
Current or former smoker	6 (42.9)	8 (80.0)	
Histological type, n (%)			0.393
NSCLC	11 (78.6)	6 (60.0)	
SCLC	3 (21.4)	4 (40.0)	
Stage, n (%)			0.358
I-II	4 (28.6)	1 (10.0)	
III-IV	10 (71.4)	9 (90.0)	
EGFR mutation, n (%)			1.000
Yes	2 (14.3)	2 (20.0)	
No	12 (85.7)	8 (80.0)	
Therapy, n (%)			0.204
Radiotherapy	11 (78.6)	5 (50.0)	
Concurrent chemoradiotherapy	3 (21.4)	5 (50.0)	

Table S3. Characteristics of patients in the discovery and validation cohorts

	Discovery cohort, (n=24)	Validation cohort 1, (n=91)	Validation cohort 2, (n=23)	P value
Gender, n (%)				<0.001
Male	18 (75.0)	7 (7.7)	14 (60.9)	
Female	6 (25.0)	84 (92.3)	9 (39.1)	
Smoking, n (%)				<0.001
Nonsmoker	10 (41.7)	91 (100.0)	5 (21.7)	
Current or former smoker	14 (58.3)	0 (0.0)	18 (78.3)	
Histological type, n (%)				<0.001
NSCLC	17 (70.8)	91 (100.0)	16 (69.6)	
SCLC	7 (29.2)	0 (0.0)	7 (30.4)	
Stage, n (%)				<0.001
I-II	5 (20.8)	2 (2.2)	NA	
III-IV	19 (79.2)	89 (97.8)	NA	
EGFR Mutation, n (%)				<0.001
YES	4 (16.7)	59 (64.8)	NA	
NO	20 (83.3)	32 (35.2)	NA	
Therapy, n (%)				<0.001
Treated	24 (100.0)	52 (57.1)	0 (0.0)	

Untreated	0 (0.0)	39 (42.9)	23 (100.0)

Note.Validation Cohort 2 comprised 23 enrolled cases (21 lung cancer patients with available saliva specimens). Comprehensive clinical metadata were analyzed for the entire cohort (n=23).
