# Supplementary figures and images for: Identification of Oral Microbiome Biomarkers Associated with Lung Cancer Diagnosis and Radiotherapy Response Prediction
Source: Pathogens. 2025 Dec 16;14(12):1294. doi: 10.3390/pathogens14121294 (PMC12735506; doi:10.3390/pathogens14121294)

A

## Wilcoxon rank – sum test bar plot on Species level

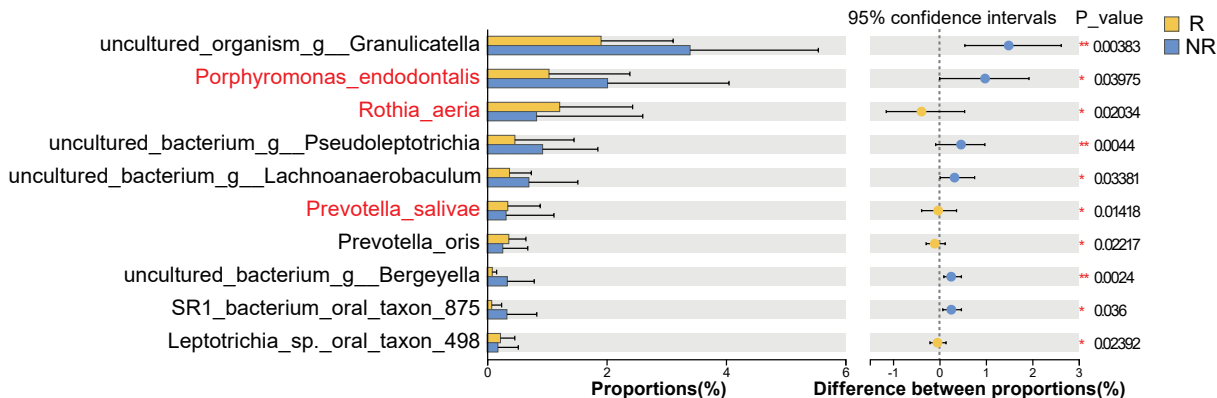

B

## LEfSe Bar

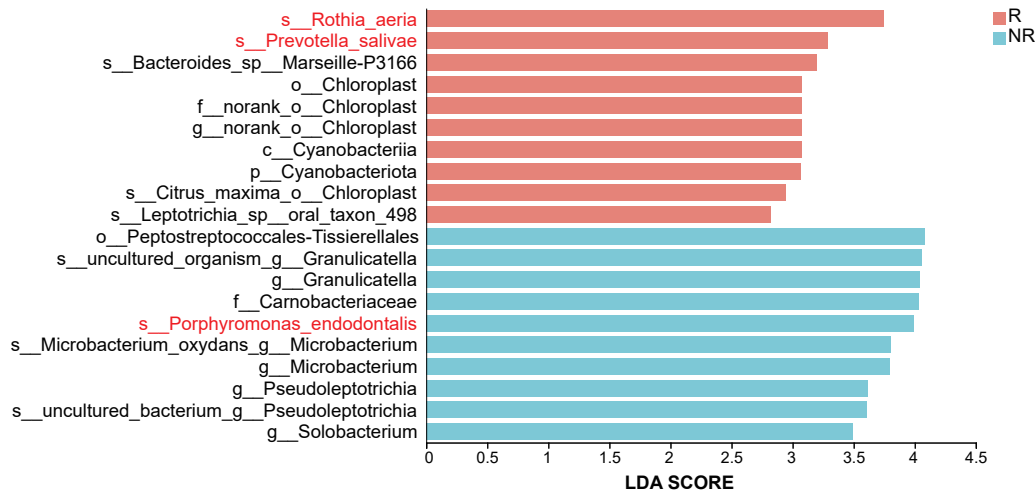

Supplement: Supplementary file 1 [file pathogens-14-01294-s001.zip › FigureS1.pdf]
